# Sulphur Case

**Published:** 1887-09

**Authors:** E. W. Sawyer

**Affiliations:** Kokomo, Ind.


### SULPHUR CASE.

E. W. Sawyer, M. D., Kokomo, Ind.

(Read at the 21st Annual Session of the Indiana Institute of Homœopathy, Indianapolis, May 25th, 1887.)

Clyde B., aged two years and eight months; about a year ago he fell out of door and cut through his scalp, and a month later his nose was stopped with bloody scabs, which disappeared in a few days. Last August there was a scab above his right ear, and the water from under it caused blisters wherever it touched the skin; about the same time had sores on face and forehead, and washing with soapsuds healed all these sores. Following the healing of these sores, not being well, Dr. L. treated him for worms, and he got better for a while, then worse again, and Dr. L. gave him more medicine, and in an hour from the first dose he had spasms. They will put him to bed, and in an hour he will be suffering terribly, apparently with his bowels, throwing himself in all directions.

The last time Dr. L. gave him worm medicine he passed some pinworms. Sometimes he sleeps on his knees and face, at other times on his back with his eyes partly open and his hands above his head, when he has these "spells." The "spells" come about half an hour apart when he has them, and last about ten minutes. The last time he had them nearly all night. Pupils first large then small during the attack. He is thirsty and drinks often, and little at a time. He is inclined to be loose in his bowels. Last six months if he runs and plays he wheezes as if he had asthma—shortness of breath.

He has an immense head and fine features; is pale and delicate looking; gave him one dose of Sulphur$^{mm}$ (Boericke & Tafel).

March 10th.—No more "spells." Gave placeb.

April 13th.—No more "spells." Warm days he gets fretful. He sleeps some in afternoons, and rests and sleeps as quietly as children of his age do at night. Bowels are about right now *and head not nearly so large as it was in the winter; a cap that rested on top of his head then comes down over his ears now.* He began to look peaked soon after taking the S.$^{mm}$ and head grows smaller, while he is growing rapidly in height, being about two inches taller than at beginning. No more signs of worms since the first and only dose of Sulphur. He has had a spell or two of sleeping on his knees and face. Doing nicely now.

India M. A., aged three years; short, stout, dark hair and eyes. Began treating her a year ago the first of this month for sore eyes, and the forty-fifth potency (Fincke) did the work promptly. Then she had a Benzoic ac. diarrhœa which it relieved at once in 2C potency, the highest I had. Then she had chills calling for Bell., which one dose of 200th cured. Last October she was threatened with typhoid, the symptoms calling for Ars., and one dose of the 40M, Jenichen's, overcame it promptly. About this time found that she was in the habit of sleeping on knees and face, and being in a hurry gave dose of Stram.$^{cm}$, which did no good. Then, discovering that she was passing too much urine that became white after standing a few minutes, and some other Phos. ac. symptoms, gave a dose of the 200th, which removed it promptly, and after that Conium mac.$^{cm}$ (Fincke) removed the disposition to sleep on knees and face.

January 15th, 1887.—Was called to see India again, and found that she had been getting along nicely until last three days, during which time she had been lying in a stupor, and had hardly been able to rouse her sufficiently to take any nourishment. She paid no attention to being handled in any way, and had the appearance of having taken an immense dose of Opium. The parents stoutly affirmed that she had taken *no* medicine, and the kind allopathic neighbors looked as though they fully expected to attend the funeral of the little girl very soon, but they knew nothing of the law of similars discovered by grand old Hahnemann. I dropped a few pellets of Opium$^{45m}$, Fincke, on her tongue, and in ten seconds, probably, she began to try to spit them out; and in less than two minutes she got down from the lap of the lady who was holding her, walked across the room to her mother and asked her for a drink of water, and has remained well since. It was funny to see the look of contempt on the neighbors' faces when the minute pellets were dropped on the child's tongue change into utter astonishment as she got up.

